# Performance Comparisons of Polymer Semiconductors Synthesized by Direct (Hetero)Arylation Polymerization (DHAP) and Conventional Methods for Organic Thin Film Transistors and Organic Photovoltaics

**DOI:** 10.3390/molecules23061255

**Published:** 2018-05-24

**Authors:** Arthur D. Hendsbee, Yuning Li

**Affiliations:** Department of Chemical Engineering and Waterloo Institute for Nanotechnology (WIN), University of Waterloo, 200 University Ave West, Waterloo, ON N2L 3G1, Canada; ahendsbe@uwaterloo.ca

**Keywords:** direct arylation, organic electronics, organic thin film transistors, organic photovoltaics, green chemistry

## Abstract

C-C bond forming reactions are central to the construction of π-conjugated polymers. Classical C-C bond forming reactions such as the Stille and Suzuki coupling reactions have been widely used in the past for this purpose. More recently, direct (hetero)arylation polymerization (DHAP) has earned a place in the spotlight with an increasing number of π-conjugated polymers being produced using this atom-economic and more sustainable chemistry. As semiconductors in organic electronics, the device performances of the polymers made by DHAP are of great interest and importance. This review compares the device performances of some representative π-conjugated polymers made using the DHAP method with those made using the conventional C-C bond forming reactions when they are used as semiconductors in organic thin film transistors (OTFTs) and organic photovoltaics (OPVs).

## 1. Introduction

Organic electronics enabled by polymer semiconductors is an exciting field of research that promises to pave the way for low-cost, flexible electronic devices such as organic thin film transistors (OTFTs) [1,2] and organic photovoltaics (OPVs) [3,4]. The performances of these devices have improved greatly in the past few years and has now become commercially viable, i.e., the field effect mobilities and the power conversion efficiencies for the polymer based OTFTs and OPVs have exceeded 10 cm^2^ V^−1^ s^−1^ [5,6,7,8] and 10% [9,10,11], respectively. While studies in this field have been more performance driven, the scale-up synthesis of polymer semiconductors has been attracting increased attention. In particular, novel synthetic methodologies that can produce high performance polymer materials in a more environmentally friendly way, i.e., using “green chemistry” [12,13,14,15], with increased atom economy and reduced production costs are highly desirable. 

Central to the construction of polymer semiconductors is the C-C bond forming reaction that links the monomeric units. Common methods for the C-C bond formation, such as Stille and Suzuki coupling reactions require an aryl halide and an aromatic compound with a reactive directing group, e.g., a boronic acid (or ester) for the Suzuki coupling and an organostannyl group for the Stille coupling. These reactions, while highly effective in C-C bond formation, require additional steps to install the directing groups, which increases the production cost and generates stoichiometric amounts of by-products that are potential health and environmental hazards. Specifically, the organotin compounds formed from the Stille coupling reactions are known to be highly toxic [16], while the boronic acid derivatives used in the Suzuki coupling reactions, which were previously assumed to be less harmful, have been recently found to be potential genotoxic hazards [17].

**Figure 1:**
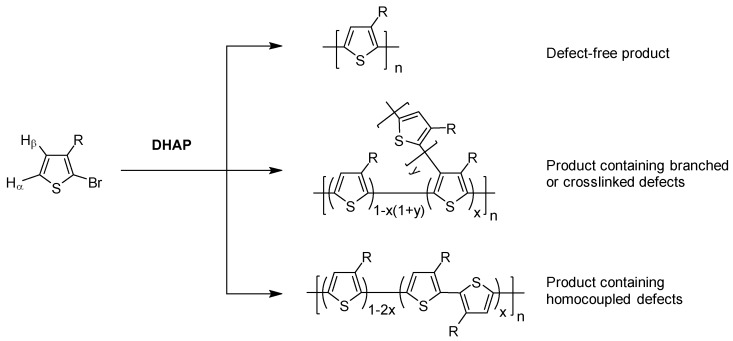
Direct (hetero)arylation polymerization (DHAP) of a 2-bromo-3-alkylthiophene, showing the potential for forming homocoupling and branching defects.

To address these issues associated with the common synthetic methods used to prepare polymer semiconductors, a novel C-C bond forming methodology, the so-called direct (hetero) arylation polymerization (DHAP) has been explored recently (Figure 1) [12]. The DHAP method eliminates the need for adding a directing group. Instead, the carbon atom with the most “active hydrogen” in the monomer is able to couple with the halogenated carbon atom in another (or the same) monomer. However, many monomer compounds have multiple C-H bonds with close dissociation energies, which can potentially be activated and react with a C-halogen bond. Furthermore, two Pd(II) complex intermediates bearing equal (hetero)aryl groups may undergo a disproportionation reaction, resulting in a homocoupling defect [18,19,20]. These side reactions may impede the formation of soluble (in the case of crosslinking side reaction) or high molecular weight (in the case of homocoupling side reaction) polymer products. Even for the polymers with good solubility and high molecular weights made by DHAP, a certain amount of branching, crosslinking, and/or homocoupling defects are frequently observed [21,22,23,24,25,26]. Figure 1 shows the formation of these defects in the DHAP of 3-alkyl-2-bromo-thiophene to poly(3-alkylthiophene). In the past few years, rigorous studies have been conducted to optimize the synthetic conditions to minimize or eliminate these side reactions. With a better understanding of the DHAP mechanism, a number of high-quality polymer semiconductors with fewer structural defects have been synthesized using the DHAP method [27].

An important question or concern from the organic electronics community is: are the performances of the polymers made by DHAP comparable to those of the polymers made by the conventional synthetic methods? In this review, we will provide a preliminary answer to this question by judiciously selecting some representative polymer semiconductors that were made by both the DHAP method and the conventional methods and compare their performances in OTFTs and OPVs. For the recent and significant progress made in clarifying the mechanism and optimizing reaction conditions of DHAP to improve the yield and molecular weight and reduce/eliminate structural defects of the resulting polymers, readers are directed to several recent review articles [12,28,29,30].

## 2. Polymer Semiconductors for Organic Thin Film Transistors

**Figure 2:**
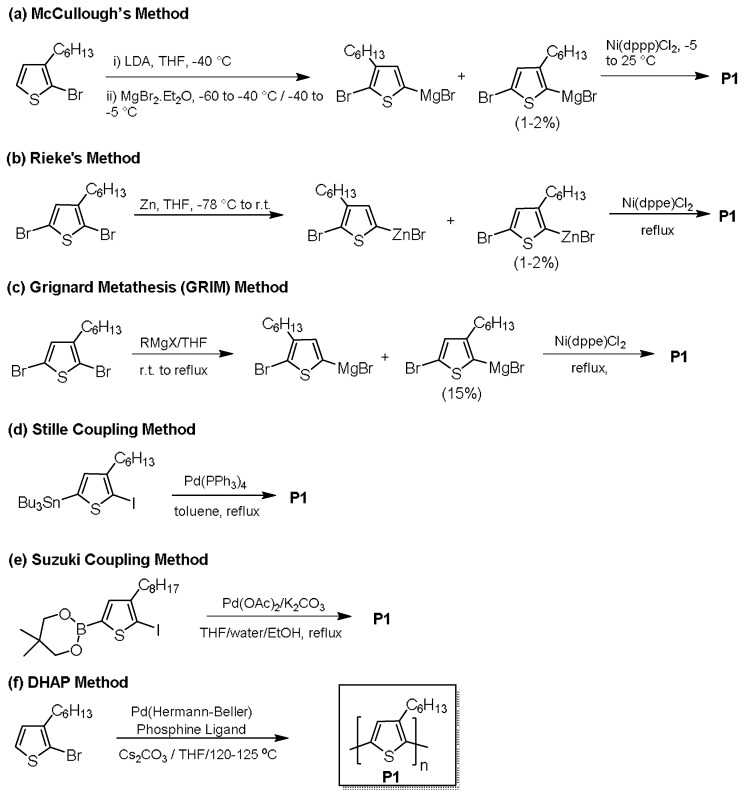
Synthesis of rr-P3HT by (**a**) McCullough’s [38], (**b**) Rieke’s [40], (**c**) Grignard metathesis (GRIM) [43], (**d**) Stille coupling [42], (**e**) Suzuki coupling [41], and (**f**) DHAP [45] methods.

Poly(3-hexyl-thiophene-2,5-diyl) (**P1**, Figure 2), also known as P3HT, is one of the most widely studied polymer semiconductors to date. Due to its widespread use in the organic electronics community, it has become a standard, to which new materials can be compared [31]. The monomer unit, 3-hexyl-thiophene-diyl, is unsymmetrical, which means that three different types of dyads, heat-to-head (HH), head-to-tail (HT), and tail-to-tail (TT) can be formed depending on the positions of the hexyl substituents on the two thiophene units. In an HH dyad, the two closely positioned hexyl substituents would cause severe twisting of the two thiophene rings, which would disrupt the π-conjugation pathway along the polymer backbone. The TT dyad is highly coplanar, but the formation of each TT dyad is accompanied with the formation of one HH dyad during the polymerization. On the other hand, the HT dyad is highly coplanar and a P3HT comprising of only HT dyads is a ‘regioregular’ HT P3HT or commonly rr-P3HT. The first P3HT was made using an FeCl_3_-mediated oxidative coupling polymerization, which contained a significant amount of HH dyads with an HT mol % or regioregularity of ~70–80% [32,33,34,35] and thus had a rather twisted backbone. P3HT made in this way has poor crystallinity, which leads to low charge carrier mobilities of ~10^−4^ cm^2^ V^−1^ s^−1^ in OTFT devices [36].

In 1992, McCullough et al. reported the use of Grignard reagents to produce an rr-P3HT with a high regioregularity of up to 98% [37,38]. Also reported in 1992 was the discovery of a synthetic methodology based on zinc reagents by Rieke et al. [39,40], which could produce rr-P3HT with regioregularity of >98%. In subsequent studies, regioregular poly(3-alkyl-thiophene-2,5-diyl) samples were also made using Suzuki coupling (HT mol % = 96–97%) [41], Stille coupling polymerization (HT mol % > 96%) [42], or Grignard metathesis (GRIM, HT mol % > 98%) [43,44]. The synthetic schemes for these methods are depicted in Figure 2. P3HT with a high HT mol % of 96% made by the Rieke method demonstrated a high hole mobility of up to 0.10 cm^2^ V^−1^ s^−1^ in OTFTs, which is several orders of magnitude higher than that for the P3HT with lower regioregularity prepared using FeCl_3_ and Yamamoto couplings [36]. Thus, it would be no exaggeration to say that the birth of rr-P3HT has brought the study of organic electronics into a new era.

The aforementioned synthetic routes to rr-P3HT require additional synthetic steps to add a directing group and/or produce stoichiometric harmful by-products. Thus, it was of interest to researchers to explore alternative synthetic pathways for forming highly regioregular P3HT without the above drawbacks.

In 1998, Lemaire and co-workers reported a new method to synthesize polythiophenes using 2-iodothiophenes as the monomers and Pd(OAc)_2_ as the catalyst [46]. The authors initially thought the polymerization was a Heck-type reaction, but it was later found to be a direct (hetero) arylation reaction, which involves a direct coupling of an active aromatic C-H bond with an aromatic C-X bond (X is halide) to form a C-C bonding motif [47]. In 2010, Wang et al. conducted a DHAP of 2-bromo-3-hexylthiophene with Herrmann’s catalyst to make an rr-P3HT [45]. High number average molecular weights (*M*_n_) of up to 31 kDa were achieved, which are much higher than those of the polythiophenes (~10^3^ Da) through DHAP reported previously by Lemaire and coworkers [46]. A very high degree of regioregularity (>98%) of this rr-P3HT was confirmed by the ^1^H NMR spectroscopic analysis, indicating that electronic grade P3HT was attainable using DHAP synthetic methods. 

Pouliot et al. [48] prepared a sample of **P1** with a very high regioregularity of >99.5% and an *M*_n_ of 33 kDa by optimizing the synthesis of the 2-bromo-3-hexylthiophene monomer and DHAP conditions (**P1_DHAP1_**, Table 1). They compared the OTFT performance of this polymer with other rr-P3HT polymers made by GRIM and Rieke’s methods using the same device configuration and testing conditions (**P1_GRIM_** and **P1_Rieke_**, Table 1). It was found that high hole mobilities of up to 0.19 cm^2^ V^−1^ s^−1^ were achieved by the polymer made by DHAP, which are much higher than those of **P1_GRIM_** (0.11 cm^2^ V^−1^ s^−1^) and **P1_Rieke_** (up to 0.02 cm^2^ V^−1^ s^−1^). The mobility trend can be explained by their regioregularity order: **P1_DHAP1_** (HT mol % > 99.5%) > **P1_GRIM_** (HT mol % = 98.0%) > **P1_Rieke_** (HT mol % = 95.5%). The results from this study show that the performance of rr-P3HT made by DHAP in OTFTs is improved compared to those of the rr-P3HT polymers made by the Rieke and GRIM methods.

**Figure 3:**
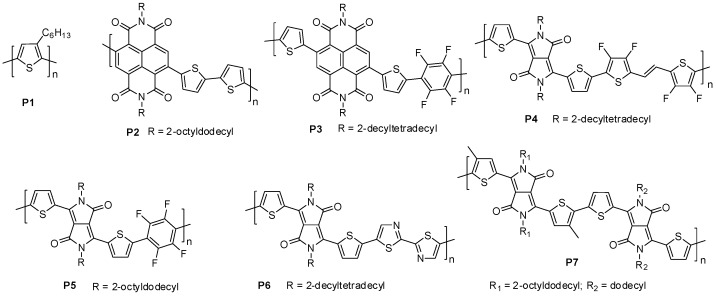
Polymers made using DHAP and conventional methods evaluated in organic thin film transistors (OTFTs).

**Figure 4:**
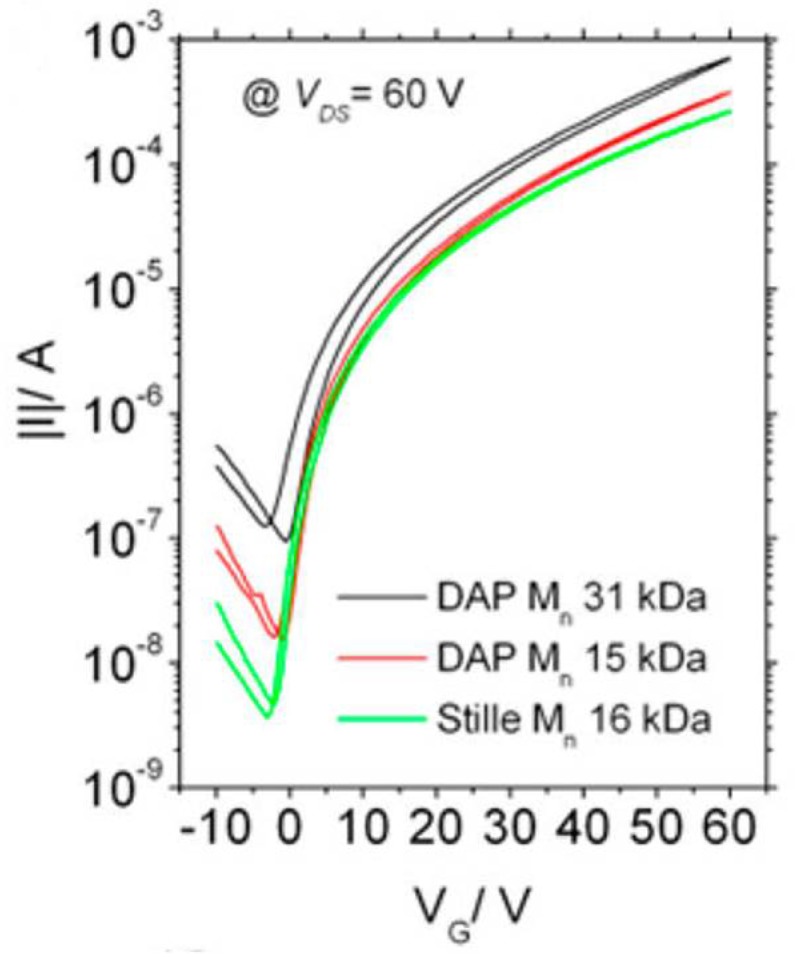
Transfer curves for transistors made from N2200 (**P2**), comparing batches from Stille and DHAP methods. Reproduced with permission from (*J. Am. Chem. Soc.* **2015**, *137*, 6705–6711). Copyright (2015) American Chemical Society.

A donor-acceptor (D-A) type polymer, poly {[*N*,*N*′-*bis*(2-octyldodecyl)-naphthalene-1,4,5,8-*bis*(dicarboximide)-2,6-diyl]-*alt*-5,5′-(2,2′-bithiophene)}, (**P2**, Figure 3) also known as N2200, is considered to be the most extensively investigated n-type polymer for OTFTs [49,50,51] and OPVs [52,53,54,55,56,57]. **P2** has commonly been synthesized using the Stille coupling polymerization [58], so the ability to synthesize it using atom-economical methods is of interest. In 2015, the DHAP synthesis of N2200 was reported by Sommer et al. [50] (**P2_DHAP_**, Table 1). Optimized DHAP conditions gave the polymer with an *M*_n_ of up to 31 kDa and a ‘perfect’ NDI-bithiophene alternating structure, with no homocoupling, branching or cross-linking, according to ^1^H NMR spectroscopic analysis. Importantly, the molecular weight of the polymer was readily controlled via in situ solvent (toluene) end capping by adjusting the monomer concentration. Another important observation was that the optical and thermal properties of the DHAP polymers reach their maxima at *M*_n_ = ~20 kDa. This is in excellent agreement with the sample produced via the Stille coupling (**P2_Stille,_** Table 1). In OTFTs, the mobilities of **P2_DHAP_** (2.9 cm^2^ V^−1^ s^−1^) and **P2_Stille_** (3.2 cm^2^ V^−1^ s^−1^) with similar *M*_n_ were very close, which are among the highest values reported for n-type polymers to date (OTFT transfer curves for **P2_DHAP_** and **P2_Stille_** are shown in Figure 4).

Another naphthalene-1,4,5,8-*bis*(dicarboximide) (NDI) based polymer **P3** (Figure 3), which is composed of NDI, thiophene and tetrafluorobenzene, was synthesized by DHAP (**P3_DHAP_, Table 1**) [59]. A rather low *M*_n_ of 7.8 kDa was obtained. A detailed ^1^H NMR spectroscopic analysis showed the absence of β-arylation, indicating that this polymer has a linear structure. Despite its rather low molecular weight, **P3_DHAP_** showed high electron mobilities of up to 1.3 cm^2^ V^−1^ s^−1^ when being used as an n-channel semiconductor in OTFTs. The same polymer synthesized by other methods has not been reported.

Diketopyrrolopyrrole (DPP) or pyrrolo[3,4-*c*]pyrrole-1,4(2*H*,5*H*)-dione is a strong acceptor building block, which has been used extensively for the construction of a large number of ultrahigh mobility donor-acceptor (D-A) type polymer semiconductors for OTFTs [60]. However, the most high-performing DPP-based polymer materials for OTFTs such as PDBT-co-TT [5,61], PDQT [62], PDPP-TVT [63], and P-29-DPPDTSE [64] have not been successfully synthesized by DHAP yet. For example, an attempt to prepare PDPP-TVT by DHAP of 3,6-*bis*(5-bromothiophen-2-yl)-2,5-*bis*(2-decyltetradecyl) pyrrolo[3,4-*c*]pyrrole-1,4-(2*H*,5*H*)-dione (DPP) and (*E*)-1,2-*bis*(thiophen-2-yl)ethane (TVT) under typical conditions failed to produce the target polymer [65]. Interestingly, when a fluorinated TVT, (*E*)-1,2-*bis*(3,4-difluorothiophen-2-yl)ethane, 4FTVT), was used, a polymer PDPP-4FTVT (**P4**) with a high *M*_n_ of up to 60 kDa was successfully obtained (**P4_DHAP_**, Table 1). This new polymer showed an ambipolar charge transport behavior with high hole and electron mobilities of up to 3.4 cm^2^ V^−1^ s^−1^ and 5.9 cm^2^ V^−1^ s^−1^, respectively, in OTFTs. This study suggests that fluorine substitution might be able to suppress the β-arylation side reactions and/or activate the α-H of the 4FTVT monomer.

Another copolymer of DPP with tetrafluorobenzene, PDPPTh2F4 (**P5**), was synthesized by DHAP [66], which has not been approached by other synthetic methods (**P5_DHAP_**, Table 1). Due to the strong electron withdrawing effect of the tetrafluorobenzene units, this polymer showed n-type electron transport semiconductor behavior, opposed to the majority of other DPP polymers that showed either p-type or ambipolar charge transport performance. High electron mobility of up to 0.60 cm^2^ V^−1^ s^−1^ was achieved in OTFTs.

A copolymer of DPP and bithiazole, PDBTz-24 (**P6**), was synthesized by Guo et al. using DHAP of dibrominated bisthienyl DPP and 2,2′-bithiazole monomers (**P6_DHAP_**, Table 1) [67]. ^1^H NMR spectra showed some unidentified structural defects possibly resulting from homocoupling and/or branching. Nonetheless, this polymer exhibited an electron transport dominant ambipolar performance with hole and electron mobilities of up to 0.06 cm^2^ V^−1^ s^−1^ and 0.53 cm^2^ V^−1^ s^−1^ in OTFTs, outperforming a similar polymer PDBTz-27 (**P6_Stille_**) with 5-decylheptadecyl side chains made by the Stille coupling previously reported by Reichmanis et al. [68], which showed electron mobilities of up to 0.31 cm^2^ V^−1^ s^−1^.

A DPP-DPP type polymer (**P7**) was synthesized via DHAP between 3,6-*bis*(thiophen-2-yl)-2,5-*bis*(dodecyl)pyrrolo[3,4-*c*]pyrrole-1,4(2*H*,5*H*)-dione and 2,5-*bis*(2-octyldodecyl)-3,6-*bis*(5-bromo-4-methylthiophene-2-yl)pyrrolo[3,4-*c*]pyrrolo-1,4(2*H*,5*H*)-dione by Pouliot et al. (**P7_DHAP_**, Table 1) [24]. A high *M*_n_ of 46 kDa along with a narrow dispersity (*M*_w_/*M*_n_) of 2.5 was obtained, which suggested that negligible side reactions occurred. P-type semiconductor performance with high hole mobilities of up to 1.2 cm^2^ V^−1^ s^−1^ was achieved in OTFTs. Interestingly, when the reactive C-H and C-Br exchanged between the two monomers, that is, 2,5-*bis*(2-octyldodecyl)-3,6-*bis*(4-methylthiophene-2-yl)-pyrrolo[3,4-*c*]pyrrolo-1,4(2*H*,5*H*)-dione and 3,6-*bis*-(5-bromothiophen-2-yl)-2,5-*bis*(2-dodecyl)pyrrolo[3,4-*c*]pyrrole-1,4(2*H*,5*H*)-dione were polymerized, the resulting polymer had a lower *M*_n_ (15 kDa) and showed lower hole mobility (0.26 cm^2^ V^−1^ s^−1^), which was ascribed to some ill-defined couplings such as homocoupling and branching.

## 3. Polymer Semiconductors for Organic Photovoltaics

The use of rr-P3HT (**P1**) synthesized by DHAP in solar cells was first reported by Thompson et al. [25]. A sample of rr-P3HT (**P1_DHAP2_**) with *M*_n_ = 19 kDa, *M*_w_/*M*_n_ = 2.0, and HT mol % = 90% and a control polymer **P1_Stille1_** with *M*_n_ = 19 kDa, *M*_w_/*M*_n_ = 2.7, and HT mol % = 93% were synthesized by DHAP and Stille coupling methods, respectively (Table 2). Interestingly, it was shown that **P1_DHAP2_** had ~0.75% β-defects, but still showed even better solar cell performance compared to **P1_Stille1_**, which had no β-defects (PCE: 2.70% for **P1_DHAP2_** vs. 2.30% for **P1_Stille1_**). The authors made another sample using DHAP, having 1.41% β-defect concentration, and found that the performance was poorer due to formation of unfavorable morphology when blended with fullerene and the low space-charge-limited current (SCLC) mobility, which resulted in low J_SC_. The authors concluded that rr-P3HT synthesized by DHAP with β-defect concentrations below 1% were able to achieve similar or better performance in solar cell devices when compared with their counterparts made by the Stille coupling method. 

Another recent study by Thompson et al. compared the properties of rr-P3HT synthesized using a “green solvent assisted” DHAP (**P1_DHAP3_**) with a sample prepared by the traditional Stille coupling method (**P1_Stille2_**, Table 2) [69]. **P1_DHAP3_** had *M*_n_ = 20 kDa, *M*_w_/*M*_n_ = 2.1, and HT mol % = 96.2%, while **P1_Stille2_** had *M*_n_ = 18 kDa, *M*_w_/*M*_n_ = 2.4, and HT mol % = 92.9%. These authors found that the use of a bulky carboxylic acid additive, neodecanoic acid, and a “green” solvent, 2-methyl-tetrahydrofuran, was critical in completely avoiding the formation of β-defects. In UV-Visible absorption spectra, the β-defect free **P1_DHAP3_** displayed a higher extinction coefficient at the wavelength of maximum absorbance (λ_max_) and stronger vibrational fine structure, indicating more structural ordering in the solid state. **P1_DHAP3_** also had larger crystalline coherence lengths, as confirmed by X-ray diffraction, which were correlated to the increased SCLC hole mobility for this polymer. When evaluated as a donor with PC_61_BM as the acceptor in solar cells, **P1_DHAP3_** outperformed **P1_Stille2_** (PCE: 3.28% vs. 2.86%, Table 2). This is in agreement with the previous results by the same group, implying that a rr-P3HT sample with negligible β-defects produced by DHAP can outperform its counterpart made by the Stille coupling [25].

**Figure 5:**
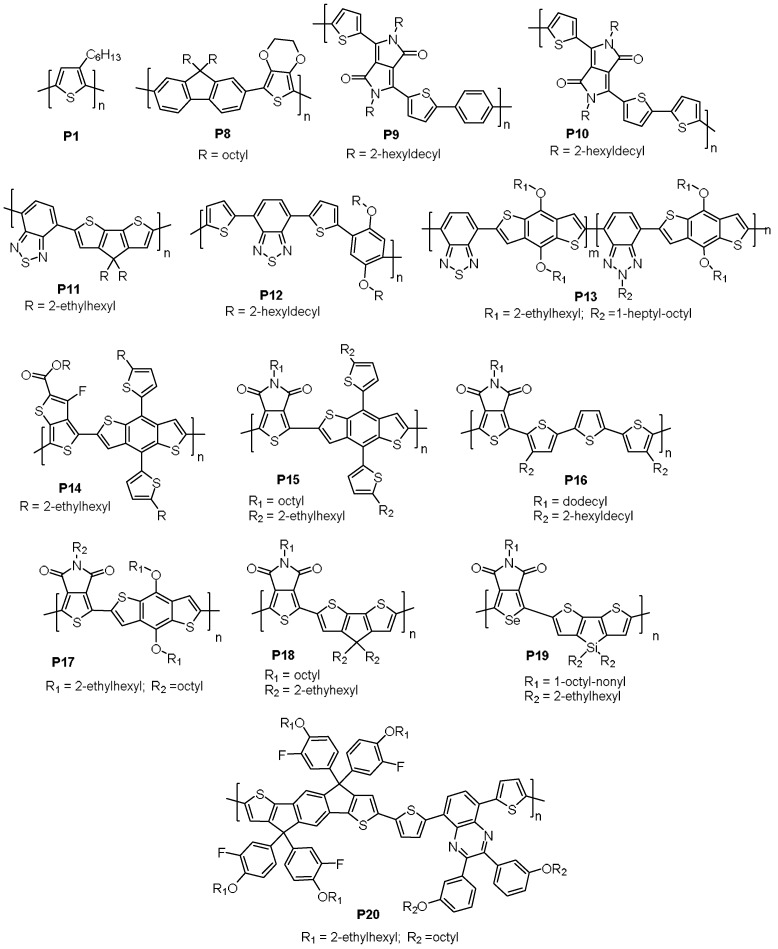
Materials made using DHAP and conventional methods, evaluated in organic photovoltaic (OPV) devices.

Kanbara et al. synthesized copolymers of fluorene and EDOT (**P8**, Figure 5) using both DHAP and Suzuki coupling methods (**P8_DHAP_** and **P8_Suzuki_**, Table 2) [70]. **P8_DHAP_** made by DHAP with a microwave reactor had a much higher *M*_n_ of 150 kDa than that of **P8_Suzuki_** made by Suzuki coupling, which had an *M*_n_ of 17 kDa. As a donor, **P8_DHAP_** demonstrated a high PCE of 4.08%, while **P8_Suzuki_** exhibited a very low PCE of 0.480%. The large discrepancy in the OPV performance of these two polymers is ascribed mainly to their different hole mobilities. In OTFTs, **P8_DHAP_** showed an average hole mobility of 1.2 × 10^−3^ cm^2^ V^−1^ s^−1^, while the average hole mobility of **P8_Suzuki_** is about two orders of magnitude lower at 3.2 × 10^−5^ cm^2^ V^−1^ s^−1^. Another **P8_DHAP_** with a lower *M*_n_ of 48 kDa showed a lower PCE of 2.55%, which was believed to be due to the presence of terminal Br groups and residual Pd impurities in the polymers, causing a decrease in the hole mobility (7.7 × 10^−4^ cm^2^ V^−1^ s^−1^). In a later study by the same group, **P8_DHAP_** showed further improved PCE of up to 4.60% after the terminal Br groups were removed through a post-polymerization treatment of the polymer with sodium *N*,*N*-diethyldithiocarbamate [71].

D-A polymers are among the most popular materials for use in OPVs and synthesis of high-quality D-A polymers by DHAP was therefore explored. Russel et al. reported on two D-A polymers based on DPP, DPP-*co*-phenylene (PDPP-TPT, **P9**, Figure 5) and DPP-co-thiophene, (PDPP3T, **P10**), which were synthesized by DHAP (**P9_DHAP_** and **P10_DHAP_**, Table 2) [22]. Optimized DHAP conditions provided a sample of **P9_DHAP_** with an *M*_n_ of 14 kDa and an *M*_w_/*M*_n_ of 1.8. **P9_DHAP_** produced via DHAP in this work was compared with one produced by Janssen’s group using a Suzuki coupling protocol (**P9_Suzuki1_**, Table 2) [72], which showed a bimodal GPC trace with two peak molecular weights of 65 kDa and 10 kDa due to the gelation and aggregation. As a donor, **P9_DHAP_** showed slightly lower PCE in OPVs than **P9_Suzuki1_** when PC_61_BM was used as the acceptor (4.37 vs. 5.50%, Table 2). Later, optimization of the Suzuki coupling reaction gave a sample of **P9_Suzuki2_** with a higher *M*_n_ reaching 72 kDa and an *M*_w_/*M*_n_ of 1.98. Solar cells using **P9_Suzuki2_** gave PCE of up to 7.40% [73]. For the second polymer reported in this work [22], PDPP3T, optimized DHAP conditions gave a sample of **P10_DHAP_** with *M*_n_ 29 kDa and an *M*_w_/*M*_n_ of 3.8. A previous paper by Janssen et al. in 2009 [74] reported on the synthesis of PDPP3T using a Suzuki coupling protocol, which gave **P10_Suzuki_** with an *M*_n_ of 54 kDa, nearly twice that of **P10_DHAP_** reported in this work. In OPVs, **P10_DHAP_** was also outperformed by **P10_Suzuki_** (4.01% vs. 4.69%, Table 2). A later work by Janssen et al. improved the synthesis of **P10** via Stille coupling [73] (**P10_Stille_**, Table 2), achieving an improved *M*_n_ of 150 kDa and an *M*_w_/*M*_n_ of 2.72. Solar cells made using **P10_Stille_** gave much better PCE of up to 7.10% when used as the donor material in OPVs. ^1^H NMR spectroscopy was unable to determine the presence of homocoupling defects in **P9_DHAP_** and **P10_DHAP_** due to the tendency to aggregate in solution; however, the authors suggested that some homocouplings (<5%) may be present in these samples because a broadening of the low energy absorption shoulders was seen, a feature which is often attributed to minor homocoupling defects [75]. Both the presence of the homocoupling defects as well as the lower molecular weights might account for the lower mobilities observed for **P9_DHAP_** and **P10_DHAP_** compared with their counterparts made by the Suzuki and Stille couplings, **P9_Suzuki1_**, **P9_Suzuki2_**, **P10_Suzuki_** and **P10_Stille_** which contained lesser amounts of defects and had higher molecular weights.

Poly[4,4-*bis*(2-ethylhexyl)-4*H*-cyclopenta[2,1-*b*;3,4-*b*′]dithiophene-2,6-diyl-*alt*-2,1,3-benzothiadiazole-4,7-diyl] (PCPDTBT, **P11**) is a high performance polymer semiconductor, used commonly as a donor in OPVs [76,77,78], and a p-type semiconductor in OTFTs [79]. In 2012, Horie et al. synthesized PCPDTBT using both DHAP and Suzuki methods (**P11_DHAP_**, **P11_Suzuki_**, Table 2) for a direct comparison [80]. **P11_DHAP_** had an *M*_n_ of 72 kDa with an *M*_w_/*M*_n_ of 4.52, while **P11_Suzuki_** had a much lower *M*_n_ of 15 kDa with an *M*_w_/*M*_n_ of 2.1. By using MALDI-TOF and ^1^H NMR spectroscopic analysis, it was found that **P11_DHAP_** contained both homocoupling and branching defects, while **P11_Suzuki_** did not. The β-defect concentration was found to be 12–13% for **P11_DHAP_**, which is well above the “threshold” value of ~1% for rr-P3HT for achieving good OPV performance observed by Thompson et al. [25]. Despite such high β-defect concentration, **P11_DHAP_** exhibited higher PCE in solar cells using PC_71_BM as the acceptor under the same processing and testing conditions compared to **P11_Suzuki_** (PCE: 3.98% vs. 3.74%. respectively) [80]. **P11_Stille_** synthesized by the Stille coupling previously, which had an *M*_n_ of 28 kDa, achieved a lower PCE of 3.50% [77] (**P11_Stille_**, Table 2). These results suggest that the PCPDTBT system has a high tolerance for β-defects, which might be due to the beneficial effect of the branching structure on the morphology of the **P11_DHAP_**:PC_71_BM blend films [80] as well as the much higher intrinsic hole mobility of this polymer [79] compared to P3HT. The fact that **P11_DHAP_** showed the best PCE is most likely due to its highest molecular weight, which is a more dominant factor than the β-defect concentration in the OPV performance. Given that the rigorous optimization of device fabrication for commercial samples of **P11** afforded an improved PCE of up to 6% when paired with PC_71_BM [76,78], it is possible that **P11_DHAP_** may also exhibit a higher PCE when the active layer formation is further optimized. 

Thompson et al. synthesized benzothiadiazole-*co*-benzodithiophene polymers (**P12**) using DHAP and Stille methods (**P12_DHAP_**, **P12_Stille2-LMW_**, **P12_Stille1-HMW_**, Table 2) [81]. The optimized DHAP protocol afforded a ‘defect-free’ PPDTBT sample **P12_DHAP_** with an *M*_n_ of 15 kDa and an *M*_w_/*M*_n_ of 2.1 (**P12_DHAP_**, Table 2). The use of THF as a solvent and the bulky neodecanoic acid as an additive was found to be essential in minimizing structural defects during DHAP based on the ^1^H NMR spectroscopic analysis [69]. Two PPDTBT samples having *M*_n_ of 59 and 16 kDa and *M*_w_/*M*_n_ of 3.3 and 2.1, respectively, were synthesized by the Stille method for comparison (**P12_Stille1-HMW_** vs. **P12_Stille2-LMW_**, Table 2). **P12_DHAP_** showed a better PCE (3.40%) compared with the counterpart **P12_Stille2-LMW_** made by Stille (PCE = 2.90%), which has a similar *M*_n_ and *M*_w_/*M*_n_. On the other hand, polymer **P12_Stille1-HMW_** with a high molecular weight made by the Stille method was able to achieve a higher PCE of 3.80%. These results illustrate, once again, the importance of the polymer molecular weight on device performance. It can be concluded that the quality of the polymer **P12** made by DHAP is similar to that made from Stille coupling if the molecular weight can be matched.

In 2015, Farinola et al. reported on the DHAP synthesis of a random copolymer (**P13**), which used benzo[*c*][1,2,5]thiadiazole or benzo[*d*][1,2,3]triazole as the accepting units and benzo[1,2-*b*:4,5-*b*′]dithiophene as the donor component [23]. Samples of **P13** were prepared via DHAP and also by Stille coupling polymerization for direct comparison. DHAP yielded a sample (**P13_DHAP_**) with a low *M*_n_ of only 10 kDa compared to the *M*_n_ of 20 kDa obtained for **P13_Stille_** synthesized via Stille coupling. In addition, the *M*_w_/*M*_n_ was much larger for the DHAP synthesized sample compared to the one made using a Stille coupling (*M*_w_/*M*_n_: 7.6 for **P13_DHAP_** vs. 3.1 for **P13_Stille_**, Table 2). A hypsochromic shift in the absorption spectra is noticed for the DHAP synthesized polymer, indicative of a lower effective conjugation length. ^1^H NMR analysis revealed the presence of β-defects for **P13_DHAP_**. When the two polymers were tested in solar cells with PC_71_BM as the acceptor, **P13_DHAP_** gave a lower PCE of 2.80% compared to a value of 4.80% for **P13_Stille_**. Such a large difference in performance between **P13_DHAP_** and **P13_Stille_** can be directly ascribed to the lower molecular weight and higher defect concentration in the former. Optimization of the catalytic systems for DHAP such as the use of bulky carboxylic acid additives and specialized catalysts [27,69,82] may provide a sample of **P13** with a lower concentration of defects and thus a higher effective conjugation length, which would improve its performance to be on par with **P13_Stille._**

**Figure 6:**
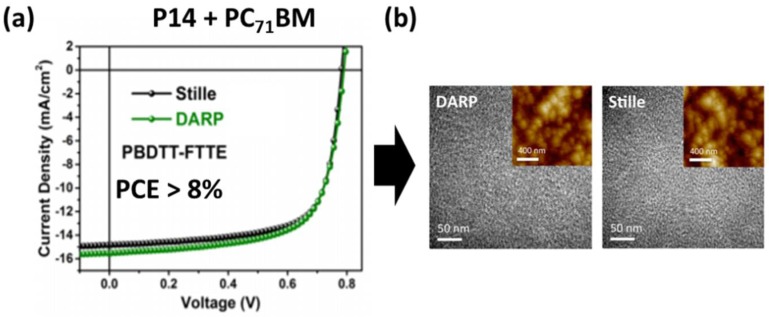
(**a**) J-V curves for Stille and DHAP produced PBDTT-FTTE with PC_71_BM; (**b**) AFM Images comparing the morphology of Stille and DHAP samples of **P6**, blended with PC_71_BM. Reproduced with permission from (*J. Am. Chem. Soc.* **2016**, *138*, 15699–15709). Copyright (2016) American Chemical Society.

In 2016, Marks et al. reported a comprehensive study to compare the physical and electronic properties of a series of D-A polymers made using both DHAP and Stille methods [83]. The first polymer is PBDTT-FTTE (**P14**), a well-known high performance p-type polymer that has been used in record setting OPVs [84,85]. It was found that increasing the steric bulkiness of the carboxylic acid additive, i.e., the use of 2,2-diethylhexanoic acid instead of pivalic acid, in the DHAP system was critical in forming high molecular weight, defect-free polymers, which is in agreement with other recent reports [69,81]. Optimization of DHAP conditions gave **P14_DHAP_** with *M*_n_ = 25 and *M*_w_/*M*_n_ = 2.2, a very close match with **P14_Stille_** made by the Stille coupling (*M*_n_ = 25 kDa and *M*_w_/*M*_n_ = 2.2). When evaluated in solar cell devices with PC_71_BM as the acceptor, **P14_DHAP_** gave an almost same PCE compared with **P14_Stille_** (PCE: 8.36% vs. 8.40%, Table 2 and Figure 6a). In addition, the two polymers showed very similar film morphologies when blended with PC_71_BM (Figure 6b). Two other pairs of D-A polymers based on thieno[3,4-*c*]pyrrole-4,6-dione (TPD), namely PBDTT-TPD (**P15_DHAP_** and **P15_Stille_**, Table 2) and PTPD3T (**P16_DHAP_** and **P16_Stille_Table 2**), were also made using both DHAP and Stille methods [83]. It was found that **P15_DHAP_** has a higher *M*_n_ than that of **P15_Stille_** (*M*_n_: 30 kDa vs. 15 kDa), while **P16_DHAP_** had a lower *M*_n_ than that of **P16_Stille_** (*M*_n_: 19 kDa vs. 30 kDa). ^1^H NMR spectroscopy confirmed that the structural regularity of both polymers made by DHAP was similar to that of the ones made by the Stille method. In addition, the absorption spectra of each pair of polymers are nearly identical. In OPVs, **P15_DHAP_** was found to outperform **P15_Stille_** (PCE: 5.84% vs. 5.20%), while **P16_DHAP_** performed slightly poorer than **P16_Stille_** (PCE: 7.20% vs. 7.38%). Since **P15_DHAP_** and **P16_DHAP_** did not contain noticeable structural defects, the differences in their OPV performance with their counterparts **P15_Stille_** and **P16_Stille_** are likely related to their different molecular weights, with the higher molecular weight ones showing higher OPV performance.

PBDTTPD (**P17**) is another high performance donor polymer for OPVs [86]. Farinola et al. conducted a study comparing the optoelectronic properties and device performance of PBDTTPD made by both DHAP and Stille methods (**P17_DHAP_** and **P17_Stille_**, Table 2) [87]. Optimized DHAP conditions produced **P17_DHAP_** with a low *M*_n_ of 12 kDa, while **P17_Stille_** synthesized via the Stille coupling protocol had an even lower *M*_n_ of 10 kDa. The slightly higher *M*_n_ of **P17_DHAP_** resulted in a red-shifted UV-Visible absorption profile as well a clearer vibronic structure in the low-energy region compared to **P17_Stille_**. In addition, the aggregation propensity of **P17_DHAP_** in solution was noted to be higher. Furthermore, I t was found that **P17_Stille_** allowed the formation of large micro-sized domains of PC_71_BM in the bulk-heterojunction blend, while **P17_DHAP_** did not. OPV devices using these two polymers as the donors and PC_71_BM as the acceptor were fabricated and compared. PCEs of up to 5.31% and 4.82% were obtained for **P17_DHAP_** and **P17_Stille_**, respectively, which is in good agreement with the disparity in their molecular weights.

A copolymer of TPD and cyclopenta[2,1-*b*:3,4-*b*′]dithiophene (**P18**) was synthesized by Kanbara et al. using an improved DHAP in toluene, which had an *M*_n_ of 25 kDa with an *M*_w_/*M*_n_ of 1.9 (**P18_DHAP_**, Table 2) [19]. The molecular weight is much higher than that (*M*_n_ = 2.5 kDa) of the same polymer made by DHAP by Harris et al. using DMAc as the solvent, where severe homocoupling occurred, resulting in the lower *M*_n_ [80]. On the other hand, the ^1^H NMR and MALDI-TOF-MS analysis of **P18_DHAP_** made in toluene in this work showed no detectable homocoupling defects. A high PCE of up to 6.80% was achieved when **P18_DHAP_** was used as a donor in OPVs, which is higher than that (PCE = 5.20%) of **P18_Stille_** prepared by the Stille coupling, which had a lower *M*_n_ of 15 kDa [88].

Ahmed et al. reported on another series of polymers based on [3,4-*c*]pyrrole-4,6-dione derivatives with sulfur, oxygen or selenium heteroatom substitution, made by the DHAP method [89]. The champion polymer from their study, **P19_DHAP_**, was based on a seleno[3,4-*c*]pyrrole-4,6-dione acceptor and a dithienosilole donor and was obtained with an *M*_n_ of 29 kDa and an *M*_w_/*M*_n_ of 1.6. When paired with PC_61_BM in inverted organic solar cells, a high PCE of 7.13% was achieved. No equivalent polymer to **P19_DHAP_** made by a conventional coupling method was reported for comparison.

A recent report by Li et al. compared the properties of indacenodithiophene (IDT) and thiophene-quinoxaline-thiophene (TQ) derived polymers (**P20**) made using DHAP and Stille coupling methods (**P20_DHAP_** and **P20_Stille_**, Table 2) [21]. **P20_DHAP_** had an *M*_n_ of 27 kDa with an *M*_w_/*M*_n_ of 1.6, while **P20_Stille_** had an *M*_n_ of 23 kDa and an *M*_w_/*M*_n_ of 1.5. The close number average molecular weights of the two samples allow for a fair comparison between the two polymers. ^1^H NMR spectroscopy, MALDI-TOF MS, and elemental analysis revealed that **P20_Stille_** contained a certain amount of TQ−TQ homocoupling defects, while **P20_DHAP_** had a very well-defined IDT-TQ alternating structure with no detectable structural defects. Interestingly, SCLC experiments showed a weak correlation between their hole mobility and structural regularity. Furthermore, grazing incidence X-ray diffraction (GIXRD) studies indicated no significant differences in the film crystallinity between two polymers. **P20_DHAP_** and **P20_Stille_** achieved PCE of 5.10% and 4.82%, respectively. It was found that the larger number of defects in the backbone of **P20_Stille_** had influenced the morphological properties and SCLC mobilities, which caused severe recombination and thus the lower PCE.

## 4. Conclusions

Recently, direct (hetero)arylation polymerization (DHAP) of a halogenated (hetero)aryl compound with another (hetero)aryl compound possessing activated C-H bonds has emerged as an alternative approach to the conventional C-C bond formation methods such as Stille and Suzuki coupling polymerization for the synthesis of linear π-conjugated polymers. Since DHAP does not require the use of a (hetero)aryl compound possessing a reactive directing group such as an organostannyl group in the case of a Stille coupling polymerization, fewer steps are needed for DHAP, which can improve the atom economy and reduce the cost for the synthesis of conjugated polymers. Furthermore, the by-product of a DHAP is a hydrogen halide (HX), which is much easier to treat compared with the toxic wastes generated from the Stille and Suzuki coupling polymerization. Therefore, DHAP has been considered a more cost-effective and “greener” technique for the mass production of conjugated polymers. However, since many (hetero)aryl compounds have more activated C-H bonds than required for the formation of a linear polymer, side reactions at the unwanted C-H bonds result in branching or cross-linking structural defects, often leading to degradation of the optoelectronic properties or even the formation of unprocessable insoluble products. In addition, homocoupling between the halogenated monomer molecules produces homocoupling defects in the polymer backbone and also results in the formation of lower molecular weight polymers. The presence of these structural defects in the polymers made by DHAP potentially have impacts on the performances of these polymers in organic electronic devices.

This review presented some exemplary conjugated polymer semiconductors made by DHAP and compared their performances in OTFTs and OPVs with their counterparts synthesized by conventional synthetic methods, such as the Stille and Suzuki coupling polymerization. It can be seen that by minimizing the structural defects and improving the molecular weight of the polymers through optimization of the DHAP conditions, similar or sometimes better OTFT and OPV performances could be achieved for the polymers made by DHAP in comparison to the ones prepared by the conventional methods. With the rapid progress made in the development of novel catalyst systems and optimization of reaction conditions (solvent, temperature, reaction time, etc.), it is expected that more and more high-performance polymer semiconductors, which have been synthesized and cannot be approached by conventional methods, will be developed. Further study of the atom-economic, cost-effective, and “greener” DHAP to enable the mass production of top-performing polymer semiconductors will propel the commercialization and wide-spread applications of organic electronics.

## Figures and Tables

**Table 1:** Comparison of Polymeric Materials Used in Thin Film Transistors.

		Synthesis			OTFT Performance				
ID	Name	*M*_n_, kDa (HT mol %)	*M*_w_/*M*_n_	Yield, %	Device	u_h_/u_e_, cm^2^ V^−1^ s^−1^	I_on_/I_off_	V_th_, V	Ref.
**P1_DHAP1_**	P3HT	33 (>99.5)	1.8	96	BGBC	0.19/-	1000/-	-/-	2016 [48]
**P1_GRIM_**	P3HT	88 (98.0)	1.5	NA	BGBC	0.11/-	1000/-	-/-	2016 [48]
**P1_Rieke_**	P3HT	25 (95.5)	1.9	NA	BGBC	0.02/-	1000/-	-/-	2016 [48]
**P2_DHAP_**	N2200	31	2.9	99	TGBC	-/2.9	-/>1000	-/-	2015 [50]
**P2_Stille_**	N2200	32	5.4	100	TGBC	-/3.2	-/>1000	-/-	2015 [50]
**P3_DHAP_**	P(ThNDIThF_4_)	7.8	1.7	-	TGBC	-/1.3	-/~10^5^	-/-	2014 [59]
**P4_DHAP_**	PDPP-4FTVT	60	4.9	93	BGTC	3.4/5.9	>10^5^/>10	−1~−15/40~55	2015 [65]
**P5_DHAP_**	PDPPTh2F4	30	2.4	75	TGBC	-/0.60	-/~10^4^	-/24.5	2015 [66]
**P6_DHAP_**	PDBTz-24	18	3.8	66	TGBC	0.06/0.53	~10^6^/~10^5^	-/-	2016 [67]
**P6_Stille_**	PDBTz-27	64	3.6	90	BGTC	-/0.31	-/10^5^	-/4	2015 [68]
**P7_DHAP_**	PDPP	46	2.5	84	BGBC	1.2/-	~10^3^/-	0/-	2015 [24]

**Table 2:** Comparison of Polymeric Materials Used in Solar Cells.

		Synthesis			Solar Cells					
ID	Name	*M*_n_, kDa (HT %)	*M*_w_/*M*_n_	Yield, %	Acceptor	PCE (Best Reported)	J_SC_, mA cm^−2^	V_OC_, V	FF, %	Ref.
**P1_DHAP2_^a^**	P3HT	19 (90.0)	2.0	-	PC_61_BM	2.70	8.70	0.62	50.0	2013 [25]
**P1_Stille1_^b^**	P3HT	19 (93.0)	2.7	-	PC_61_BM	2.30	8.37	0.61	45.0	2013 [25]
**P1_DHAP3_^a^**	P3HT	20 (96.2)	2.1	74	PC_61_BM	3.28	9.40	0.59	59.1	2017 [69]
**P1_Stille2_^b^**	P3HT	18 (92.9)	2.4	68	PC_61_BM	2.86	8.34	0.59	58.1	2017 [69]
**P8_DHAP_**	PEDOTF	150	2.89	89	PC_71_BM	4.08	9.41	0.83	52.0	2014 [70]
**P8_Suzuki_**	PEDOTF	17	2.08	85	PC_71_BM	0.48	2.58	0.59	31.0	2014 [70]
**P9_DHAP_**	PDPP-TPT	14	1.8	29	PC_71_BM	4.37	13.3	0.77	41.5	2015 [22]
**P9_Suzuki1_^c^**	PDPP-TPT	65 ^a^	-	69	PC_71_BM	5.50	10.8	0.80	65.0	2010 [72]
**P9_Suzuki2_^c^**	PDPP-TPT	72	1.98	93	PC_71_BM	7.40	14.0	0.80	67.0	2013 [73]
**P10_DHAP_**	PDPP-3T	29	3.8	45	PC_71_BM	4.01	10.3	0.71	56.2	2015 [22]
**P10_Suzuki_**	PDPP-3T	54	3.15	84	PC_71_BM	4.69	11.8	0.66	60.0	2009 [74]
**P10_Stille_**	PDPP-3T	150	2.72	85	PC_71_BM	7.10	15.4	0.67	69.0	2013 [73]
**P11_DHAP_**	PCPDTBT	72	4.52	76	PC_71_BM	3.98	13.9	0.63	45.5	2012 [80]
**P11_Suzuki_**	PCPDTBT	15	2.1	83	PC_71_BM	3.74	12.7	0.64	43.8	2012 [80]
**P11_Stille_**	PCPDTBT	28	1.5	61	PC_61_BM	3.50	11.8	0.65	46.0	2007 [77]
**P12_DHAP_**	PPDTBT	15	2.1	78	PC_61_BM	3.40	10.5	0.72	45.0	2016 [81]
**P12_Stille1-HMW_^d^**	PPDTBT	59	3.3	79	PC_61_BM	3.80	11.5	0.73	45.0	2016 [81]
**P12_Stille2-LMW_^d^**	PPDTBT	16	2.1	70	PC_61_BM	2.90	8.88	0.72	46.0	2016 [81]
**P13_DHAP_**	-	10	7.6	70	PC_71_BM	2.80	5.58	0.89	56.0	2015 [23]
**P13_Stille_**	-	20	3.1	85	PC_71_BM	4.80	9.89	0.81	60.0	2015 [23]
**P14_DHAP_**	PBDTT-FTTE	25	2.2	98	PC_71_BM	8.36	15.5	0.78	68.8	2016 [83]
**P14_Stille_**	PBDTT-FTTE	25	2.2	-	PC_71_BM	8.40	14.9	0.78	72.2	2016 [83]
**P15_DHAP_**	PBDTT-TPD	30	2.7	76	PC_71_BM	5.84	10.0	0.99	57.9	2016 [83]
**P15_Stille_**	PBDTT-TPD	15	2.4	69	PC_71_BM	5.20	9.10	0.99	58.7	2016 [83]
**P16_DHAP_**	PTPD3T	19	2.0	83	PC_71_BM	7.20	13.3	0.82	66.0	2016 [83]
**P16_Stille_**	PTPD3T	30	1.8	94	PC_71_BM	7.38	13.2	0.78	71.1	2016 [83]
**P17_DHAP_**	PBDTTPD	12	-	80	PC_71_BM	5.31	10.41	0.92	56.0	2016 [87]
**P17_Stille_**	PBDTTPD	10	-	90	PC_71_BM	4.82	9.17	0.93	57.0	2016 [87]
**P18_DHAP_**	-	25	1.9	82	PC_71_BM	6.80	13.8	0.91	53.5	2016 [19]
**P18_Stille_**	-	15	1.2	52	PC_71_BM	5.20	10.0	0.88	59.0	2013 [88]
**P19_DHAP_**	PSePD3T	29	1.6	57	PC_61_BM	7.13	13.2	0.85	64.0	2015 [89]
**P20_DHAP_**	IDT-TQ	27	1.6	71	PC_71_BM	5.10	10.8	0.89	53.4	2016 [21]
**P20_Stille_**	IDT-TQ	23	1.5	64	PC_71_BM	4.82	10.4	0.89	52.1	2016 [21]

^a^ Different synthetic conditions were used. **P1_DHAP2_**: 1 mol % Pd(OAc)_2_/pivalic acid/K_2_CO_3_/DMAc/45 °C, 72 h; **P1_DHAP3_**: 1 mol % Pd_2_(dba)_3_/P(*o*-MeOPh)_3_/neodecanoic acid/Cs_2_CO_3_/MeTHF/120 °C, 12 h; ^b^ Same synthetic conditions (Pd(PPh_3_)_4_/DMF, 95 °C, 48 h) were used. Similar device fabrication procedures, except for the annealing conditions (110 °C /40 min for **P1_Stille1_** vs. 150 °C/30 min for **P1_Stille2_**), were used; ^c^ Different synthetic conditions: **P9_Suzuki1_**: 5 mol % Pd_2_(dba)_3_/11 mol % PPh_3_/4 eq. K_3_PO_4_/Aliquat 336, toluene-water (8:1)/115 °C, 72 h; **P9_Suzuki2_**: 3 mol % Pd_2_(dba)_3_/12 mol % PPh_3_/5 eq. K_3_PO_4_/Aliquat 336, toluene-water (6:1), 115 °C, 16 h; ^d^ Different monomers were used: **P12_StilleLMW_**: (5,5′-(2,5-*bis*((2-hexyldecyl)oxy)-1,4-phenylene)*bis*(thiophene-5,2-diyl))*bis*(trimethylstannane) and 4,7-dibromobenzo[*c*][1,2,5]thiadiazole; **P12_StilleHMW_**: 4,7-*bis*(5-(trimethylstannyl)thiophen-2-yl)benzo[*c*][1,2,5]thiadiazole and 1,4-dibromo-2,5-*bis*((2-hexyldecyl)oxy)benzene; ^e^ A bimodal GPC trace shows two peak molecular weights at 65 kDa and 10 kDa, respectively.
